# High-Performance Gas Sensor of Polyaniline/Carbon Nanotube Composites Promoted by Interface Engineering

**DOI:** 10.3390/s20010149

**Published:** 2019-12-25

**Authors:** Weiyu Zhang, Shuai Cao, Zhaofeng Wu, Min Zhang, Yali Cao, Jixi Guo, Furu Zhong, Haiming Duan, Dianzeng Jia

**Affiliations:** 1 School of Physics Science and Technology, Xinjiang University, Urumqi 830046, China; zweiyu0244@163.com (W.Z.); caoshuaiy@163.com (S.C.); minzhang0816@163.com (M.Z.); 2 Key Laboratory of Energy Materials Chemistry, Ministry of Education, Key Laboratory of Advanced Functional Materials, Xinjiang University, Urumqi 830046, China; caoyali@xju.edu.cn (Y.C.); jxguo1012@163.com (J.G.); zhfuru@shzu.edu.cn (F.Z.)

**Keywords:** interface engineering, polyaniline, carbon nanotubes, gas sensor, hierarchical structure

## Abstract

Inspired by the enhanced gas-sensing performance by the one-dimensional hierarchical structure, one-dimensional hierarchical polyaniline/multi-walled carbon nanotubes (PANI/CNT) fibers were prepared. Interestingly, the simple heating changed the sensing characteristics of PANI from p-type to n-type and n-type PANI and p-type CNTs form p–n hetero junctions at the core–shell interface of hierarchical PANI/CNT composites. The p-type PANI/CNT (p-PANI/CNT) and n-type PANI/CNT (n-PANI/CNT) performed the higher sensitivity to NO_2_ and NH_3_, respectively. The response times of p-PANI/CNT and n-PANI/CNT to 50 ppm of NO_2_ and NH_3_ are only 5.2 and 1.8 s, respectively, showing the real-time response. The estimated limit of detection for NO_2_ and NH_3_ is as low as to 16.7 and 6.4 ppb, respectively. After three months, the responses of p-PANI/CNT and n-PANI/CNT decreased by 19.1% and 11.3%, respectively. It was found that one-dimensional hierarchical structures and the deeper charge depletion layer enhanced by structural changes of PANI contributed to the sensitive and fast responses to NH_3_ and NO_2_. The formation process of the hierarchical PANI/CNT fibers, p–n transition, and the enhanced gas-sensing performance were systematically analyzed. This work also predicts the development prospects of cost-effective, high-performance PANI/CNT-based sensors.

## 1. Introduction

Sensors are the most basic and core components of the Internet of Things (IoT) [1]. With the rapid development of IoT and the increasing attention to air quality, the high-performance gas sensors for monitoring the indoor and outdoor air quality becomes increasingly urgent. Nitrogen dioxide (NO_2_) and ammonia (NH_3_) are typical atmospheric pollutants. Even at lower concentrations, both NH_3_ and NO_2_ can cause irritation to the eyes, skin, and respiratory system [2]. Therefore, the Occupational Safety and Health Administration (OSHA) has designated the total weight-average (TWA) permissible NH_3_ exposure limit over 8 h to be between 25 and 50 ppm [3,4], and American Conference of Governmental Industrial Hygienists (ACGIH) recommended a threshold exposure limit of 200 ppb NO_2_ [5]. As a result, the real-time and sensitive monitoring of NO_2_ and NH_3_ requires that the IoT-based gas sensors should have not only good selectivity and reversibility, but also the rapid response and high sensitivity. Therefore, gas sensors of various nanomaterials have been developed to detect effectively NO_2_ and NH_3_. In this context, Lu et al. synthesized hierarchical reduced graphene oxide (rGO)/ZnO composites by a facile solution-processed method [6]. The response of rGO/ZnO composites to 50 ppb NO_2_ reached 12, which was seven times higher than that of pristine ZnO at 100 °C. The limit of detection (LOD) could be achieved as low as 5 ppb. The enhanced sensitivity was attributed to the local p–n heterojunctions between rGO and hierarchical ZnO. However, both response time and recovery time were more than 5 min. Kim et al. produced WS2 edge functionalized carbon nanofibers with multiple tubular pores (WS2@MTCNFs) [7]. The WS2@MTCNFs exhibited a response of 15% to 1 ppm NO_2_ at room temperature (RT) compared with the pristine carbon nanofibers (2% to 1 ppm NO_2_), which was attributed to the synergistic effects originated from enhanced surface area, open porosity of MTCNFs and remarkably increased active spots on the surface from WS_2_ edge sites. Yet, the response time of WS_2_@MTCNFs was still as long as 3.73 min for 10 ppm NO_2_. Manoharet al. reported that the chemiresistive sensor of single walled carbon nanotube (SWCNT) bundles on cellulosics (paper and cloth) achieved the detection of aggressive oxidizing vapors of NO_2_ and chlorine at 250 and 500 ppb, respectively [8]. Inkjet-printed films of SWCNTs on 100% acid-free paper were significantly more robust than dip-coated films on plastic substrates. Nevertheless, both response time and recovery time were also more than 5 min. Swager et al. mechanically drew CNTs on different type of papers and used as NH_3_, showing a theoretical limit of detection of 0.36 ppm [9]. The response time and recovery time are still about 3 min. Liu et al. constructed the flexible NO_2_ sensors by spin coating the colloidal PbS quantum dots onto Al_2_O_3_, polyethylene terephthalate and filter paper, respectively [10]. The paper-based sensors displayed the highest gas-sensing response to 50 ppm NO_2_, because the higher film porosity from porous and rough nature of paper leaded to the more efficient exposure of PbS surfaces to the target gas molecules. As a result, the response time and recovery time for 50 ppm NO_2_ was shortened to about 12 s and 37 s, respectively. However, PbS contains heavy metal ions, which are harmful to the environment.

Polyaniline (PANI) has also been widely studied as promising candidates in developing electronics and sensors [11,12,13], due to the advantage of tunable morphology, scalable preparation, easy doping and low cost [14,15,16]. For example, Rutledge et al. fabricated chemiresistive sensors using electrospun PANI fibers doped with different levels of sulfonic acid [17]. The doped and undoped PANI fibers were excellently sensitive to NH_3_ and NO_2_, respectively. The electrospun PANI fibers exhibited changes in measured resistances up to 60-fold for 500 ppm NH_3_, and more than five orders of magnitude for 50 ppm NO_2_ due to the one-dimensional structure and high permeability of PANI fibers. The response times were about 50 s for exposure and 70 s for purging for 1 to 50 ppm NO_2_. However, PANI is relatively hard to process into fibers due to its rigid backbone and high incompatibility. Elastic polymers are often used in electrospinning of PANI, but the introduction of elastic polymers hinders the effective exposure of PANI, inhibiting the sensing performance. There is still big room for further improvement in sensing speed and sensitivity of PANI. Considering the good sensitivity and relative harmlessness of multi-walled carbon nanotubes (MWCNTs) and PANI, one-dimensional hierarchical PANI coated MWCNTs (PANI/CNT) composites were prepared using MWCNTs as templates, achieving the real-time and sensitive detection of NO_2_ and NH_3_. Interestingly, the sensing performance of PANI and PANI/CNTs were changed from p-type to n-type by simple heating at 80 °C for 24 h. In this way, p-type MWCNTs and n-type PANI form many local p–n heterojunctions at their core–shell interfaces, improving the sensing performance of PANI/CNTs. As far as we know, this is the first time to fabricate a one-dimensional core–shell PANI/CNT composite with p–n heterojunctions and its chemiresistive gas sensors.

## 2. Materials and Methods

### 2.1. Materials

Aniline (99%, Alfa Aesar, Beijing, China) was distilled before use and stored at 4 °C. Ammonium persulfate (98%), hydrochloric acid (HCl 38%), ethanol (C_2_H_6_O), acetone (C_3_H_6_O), formaldehyde (CH_2_O) and ammonium hydroxide were purchased from Sinopharm Chemical Reagent Co., Ltd., Beijing, China. Carbonylated MWCNTs with purity > 95% were purchased from Chengdu Institute of Organic Chemistry, China. Carbonylated MWCNTs has the 10–30 μm of length, 10–20 nm of outer diameter, and >200 m^2^/g of special surface area. NO_2_ diluted with high purity nitrogen was purchased from Dalian Special Gases Co., Ltd., Dalian, China.

### 2.2. Preparation of Sensing Materials

First, 2 mg of carbonylated MWCNTs, 8 mL of HCl and 8 mL of aniline were placed in 200 mL of deionized water and dispersed by ultrasound for 30 min. Second, 8 g of ammonium persulfate were added to 20 mL of deionized water and magnetically stirred for 5 min. Third, the two solutions were cooled by ice water mixture to 0 °C under the condition of magnetic stirring and mixed evenly by the stirring for 18 h. Finally, the PANI/CNT composites were collected by centrifugation, washing and drying at RT. PANI was also prepared with the same formulation and disposal method as PANI/CNT composites, except for the absence of MWCNTs. PANI and PANI/CNT dried at RT are p-type, and they are recorded as p-PANI and p-PANI/CNT, respectively. To evaluate the effect of heating on the gas-sensing performance, PANI and PANI/CNT were dried at 80 °C for 24 h and the products were designated as n-PANI and n-PANI/CNT, respectively.

### 2.3. Device Fabrication and Sensing Tests

First, dried samples and deionized water were mixed in a mass ratio of 1:5 and ground gently into paste. Second, the paste was then coated on a ceramic substrate by a thin brush to form a sensing film on which platinum interdigitated electrodes with both finger-width and interfinger spacing of about 200 µm was previously printed. The thickness of the sensing layer was controlled by controlling the number of coating. Third, the sensors were dried at 25 °C about 24 h to form a sensing film. To reduce the interference of the external environment, the temperature and humidity of the testing room were controlled at 25 ± 1 °C and 35 ± 2% respectively by an air conditioning system. 100 ppm NO_2_ diluted with high purity nitrogen (Dalian Special Gases Co., Ltd.) were used for sensing test. Different concentration of NO_2_ gas is prepared according to Formula (1)

Q = C × V/C_O_ (1)

where: Q and V is the volume of gas to be taken, and the volume of gas chamber (mL), respectively; C and C_O_ is the concentration of gas to be prepared and the concentration of gas source (ppm), respectively. For the volatile organic compounds of different concentrations are prepared according to Formula (2)

**Figure 1:**
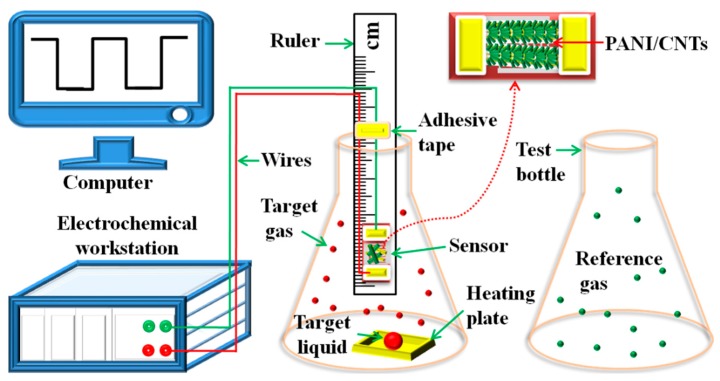
Schematic of sensing test for chemiresistive sensor of hierarchical PANI/CNT fibers.

Q = (V × C × M)/(22.4 × d × ρ) × 10 − 9 × (273 + T_R_)/(273 + T_B_) (2)

where: Q and V is the volume of the liquid to be taken and the volume of the test bottle (mL), respectively; M is the molecular weight of the substance (g); d is the purity of the liquid; C is the concentration of the gas to be prepared (ppm); ρ is the density of the liquid (g/cm^3^); T_R_ and T_B_ is the test ambient temperature and the temperature in the test bottle (°C). During the preparation of the target vapors by heating, the containers were sealed. After a certain concentration of vapors were generated, the sealed containers were placed at 25 ± 1 °C for about 2 h to reduce the temperature of the target vapors to 25 ± 1 °C. The gas-sensing performance was tested at 25 ± 1 °C by an electrochemical workstation (CIMPS-2, ZAHER ENNIUM) with a bias voltage of 4 V. As shown in Figure 1, to facilitate sensing tests, the sensor was fixed on the ruler by the metal clip and adhesive tape, so that the sensor can be stably moved into different atmosphere (target gas and reference gas) by moving the ruler from the target gas to the reference gas. This reduces the signal fluctuation caused by the moving sensor. The response in electric current is defined as, Response = ΔI/I_R_ = (I_G_ − I_R_)/I_R_ × 100%, where I_R_ and I_G_ are the electric current of the sensor in reference gas and in target gas, respectively. The response time is defined as the period in which the electric current of the sensor reaches 90% of the response value upon exposure to the target gas, while the recovery time is defined as the period in which the electric current of the sensor changes to 10% of the response value after the target gas is removed [18].

### 2.4. Characterization

To evaluate the properties of materials, PANI, MWCNTs and PANI/CNT fibers were tested by Fourier transform infrared (FTIR) spectrometer (Bruker VERTEX 70, Karlsruhe, Germany), the UV-vis spectra (UV-1800 spectrophotometer, Shimadzu, Reinach BL, Switzerland) and the X-ray photoelectron (XPS) spectroscopy (Thermo SCIENTIFIC ESCALAB 250XI, Waltham, MA, USA) measurements were carried out using the Al K alpha as the excitation source and used C1s (284.8 ev) for peak correction. Zeta potentials of samples were measured by a Zeta potentiometer (NanoBrook 90plus PALS, Holtsville, NY, USA). The morphology of PANI, MWCNTs and PANI/CNTs fibers was observed by transmission electron microscope (TEM, JEM-2100F, Japan) and field emission scanning electron microscopy (FE-SEM, S-4800, Hitachi, Japan). The electrical conductivity was measured using a digital, four-pointprobe RTS-9 resistivity measurement system at 25 °C, and the diameter and thickness of the specimens are 11.48 and 2–2.3 mm, respectively.

## 3. Results

### 3.1. Preparation of Hierarchical PANI/CNT Composite Fibers

**Figure 2:**
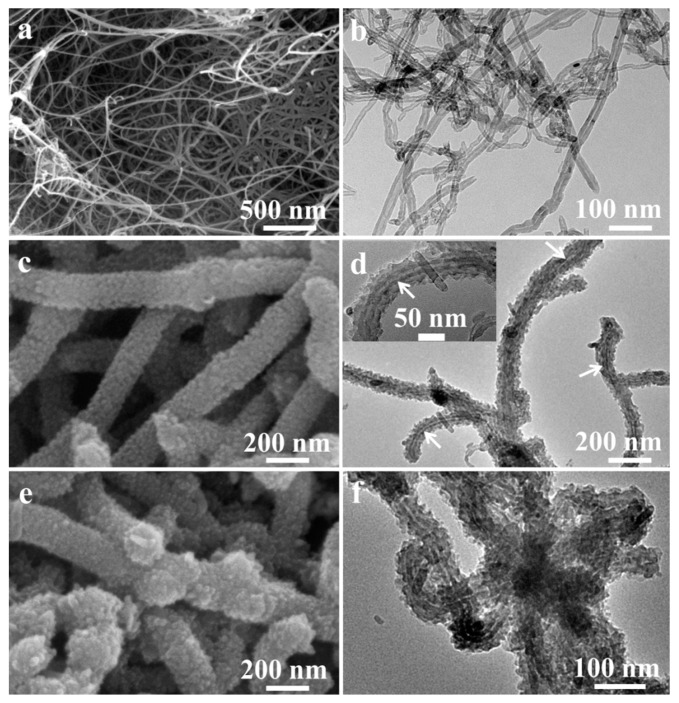
(**a**) SEM, (**b**) TEM image of MWCNTs; (**c**) SEM, (**d**) TEM image of p-PANI/CNT fibers, Inset: Magnified TEM image of p-PANI/CNT fibers; (**e**) SEM, (**f**) TEM image of n-PANI/CNT fibers.

It can be seen from the SEM and TEM images of MWCNTs (Figure 2a,b) that carboxylated MWCNTs have diameters ranging from 10 to 20 nm and lengths of about several microns. One can see that the hierarchical p-PANI/CNT composites are homogeneous and fibrous from SEM and TEM images in Figure 2c,d. Hierarchical p-PANI/CNT fibers have diameters ranging from 100 to 200 nm. As shown in Figure S1a,b the zeta potentials of carboxylated MWCNTs and aniline monomers are opposite, about−30 and 40 mV, respectively. The zeta potentials of the mixture of carboxylated MWCNTs and aniline monomers was also measured immediately after ultrasonic dispersion, showing two almost opposite potentials (Figure S1c). After 3 min, only one potential about 25 mV was left (Figure S1d), implying that aniline monomers were adsorbed on carboxylated MWCNTs because of the electrostatic interaction between amino group and carboxyl group and π-π conjugation interactions between benzene rings (Figure S2) [18]. Therefore, in the presence of oxidants, aniline monomers adsorbed on MWCNTs were polymerized to PANI using MWCNTs as template, forming the hierarchical p-PANI/CNT fibers. The morphology of n-PANI/CNT composites evolved from p-PANI/CNT fibers dried at 80 °C for 24 h did not change compared with the p-PANI/CNT fibers (Figure 2e,f) and one-dimensional hierarchical structure was well preserved. In addition, the PANI fibers were prepared with the same formulation except for the absence of MWCNTs in order to compare the gas-sensing performance and the TEM image is shown in Figure S3.

**Figure 3:**
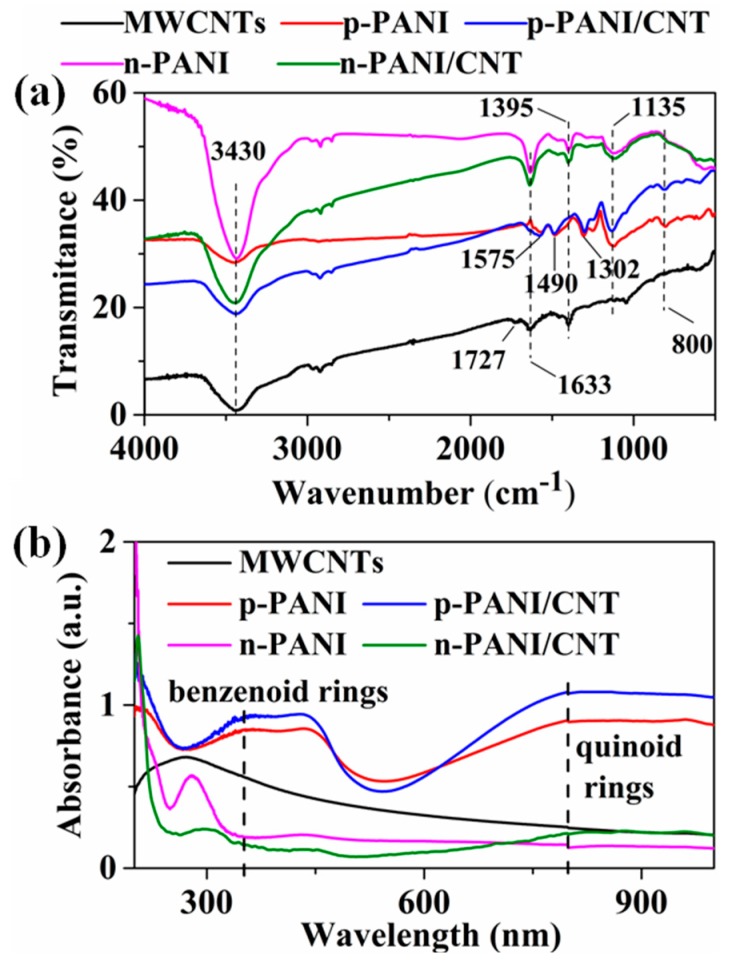
(**a**) FTIR spectra, (**b**) UV-vis spectra of MWCNTs, p-PANI, p-PANI/CNT, n-PANI and n-PANI/CNT fibers.

Figure 3a,b shows the FTIR spectra of samples. For the MWCNTs, the peaks around 3430, 1727 and 1395 cm^−1^ are attributed to the O-H, C=O in COOH, and C-O in C-O-C functional groups, respectively [19]. The peak at 1633 cm^−1^ is attributed to the H-bonded C=O at the surface of MWCNTs [20]. For p-PANI and p-PANI/CNT composites, the peaks centered at 1575, 1490, 1302, 1135 and 800 cm^−1^ were attributed to the vibration of C=N stretching of quinoid rings, C=C stretching of benzenoid rings, C-N, N=Q=N (Q representing the quinoid ring) and C-H of PANI, respectively [19,21,22]. The disappearance of the peak at 1727 cm^−1^ in the p-PANI/CNT composite indicates that the MWCNTs are uniformly coated by PANI. The ratio (I_1575_/I_1490_) of quinoid to benzenoid ring determined by FTIR observed in p-PANI, p-PANI/CNT were 1.01, 0.99, respectively. For n-PANI and n-PANI/CNT, the peaks of quinoid and benzenoid ring almost disappeared. After heat treatment, the FTIR spectra of samples changed and the new peaks at 1395 and 1633 cm^−1^ appears, which is similar to the carboxylated MWCNTs prepared by oxidation. This shows that the PANI is partially oxidized during the heat treatment process, forming the C-O-C and H-bonded C=O. PANI does not contain oxygen in the structural formula. However, it can be seen from the XPS data that there are a lot of oxygen elements in the samples after heat treatment (Figure S4), which indicates that PANI may be oxidized to some extent during the heat treatment process. In addition, the almost disappeared C-H peak at 800 cm^−1^ also indicates the partial oxidation of PANI after heat treatment. In other words, heat treatment has destroyed the structure of PANI to some extent. As shown by the UV-vis spectra in Figure 3b, the p-PANI and p-PANI/CNT composites display the similar absorptive characteristics. The peaks at 345 and 800 nm are attributed to benzenoid rings and quinoid rings, respectively [21,23,24]. The quinoid/benzenoid ratio in p-PANI and p-PANI/CNT is about 1.2 and 1.1, respectively. For the n-PANI and n-PANI/CNT, the characteristic peaks at 345 and 800 nm almost disappeared, which is consistent with the FTIR results. This also indicated that during heat treatment, prolonged heating may destroy the structure of PANI, which results in changes in properties of samples. This can also be reflected in changes of their conductivity and their elemental composition. Compared with p-PANI (1.4 × 10^−3^ s/cm), the conductivity of p-PANI/CNT increased by 5.5 times (Table S1). This can be attributed to strong π-π interactions between the MWCNTs and PANI in which the MWCNTs with the high conductivity (45.4 s/cm) enhances the p-PANI through charge transfer at the interface [21]. After heat treatment, the conductivity of p-PANI and p-PANI/CNT decreased from 1.4 × 10^−3^ and 9.3 × 10^−3^ s/cm to 8.7 × 10^−6^ and 1.5 × 10^−6^ s/cm of n-PANI and n-PANI/CNT, respectively (Table S1). It is worth noting that the conductivity of n-PANI/CNT is much lower than that of n-PANI. When p-PANI is transformed into n-type semiconductors, the local p–n heterojunctions form at the interface between n-type PANI and p-type MWCNTs. Thus, the electrons in n-type PANI combine with the holes in p-type MWCNTs at the local p–n heterojunctions, resulting in the lower carrier concentration of n-PANI/CNT [25,26,27]. In addition, the band gaps of samples have been roughly estimated according to the plot in Figure S5, which is obtained via the transformation based on the UV-vis spectra. The estimated band gaps of p-PANI, p-PANI/CNT, n-PANI and n-PANI/CNT are approximately 2.42, 2.22, 2.44, 3.58 and 3.37 eV, respectively. This shows that heat treatment does have a significant effect on the band gaps of the samples. Combined with the data of UV-Vis absorption and gas-sensing, we speculated that oxidation may destroy the structure of PANI to a certain extent, and then cause the change of structure and properties of PANI. The change of surface properties and carrier concentration has a significant effect on the gas-sensing properties of materials, which is confirmed by their gas performance.

### 3.2. Gas-Sensing Performance of PANI/CNTs Composite Fibers

**Figure 4:**
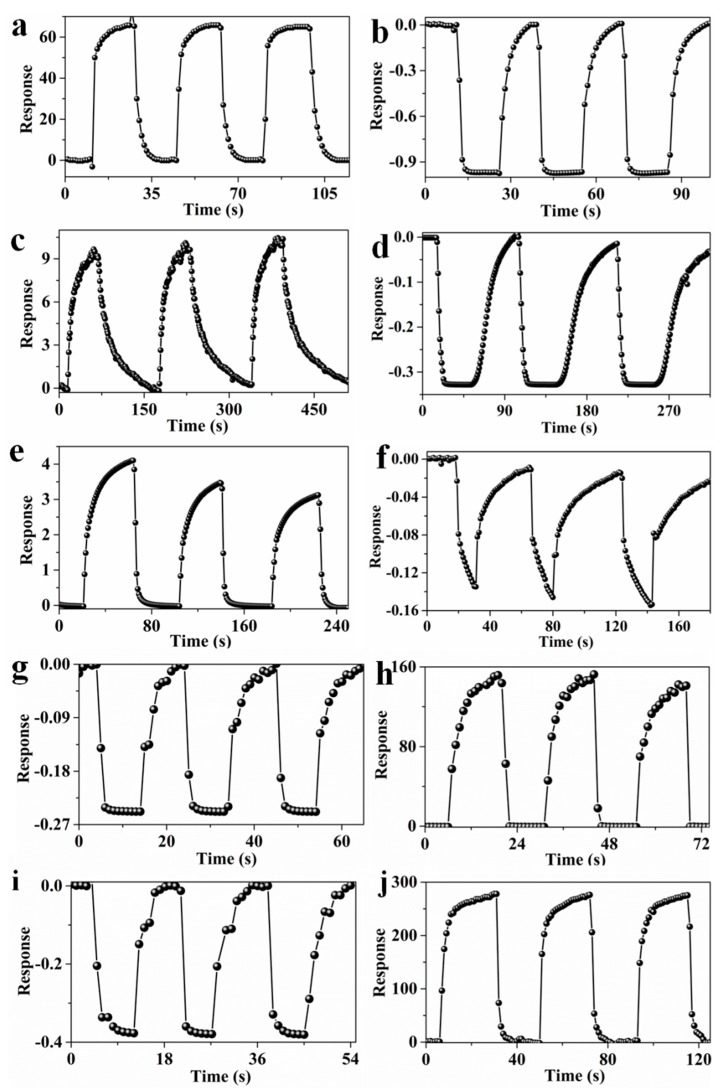
Response curves of p-PANI/CNT to 50 ppm of (**a**) NO_2_, (**b**) NH_3_; Response curves of p-PANI to 50 ppm of (**c**) NO_2_, (**d**) NH_3_; Response curves of MWCNTs to 50 ppm of (**e**) NO_2_, (**f**) NH_3_; Response curves of n-PANI to 50 ppm of (**g**) NO_2_, (**h**) NH_3_; Response curves of n-PANI/CNT to 50 ppm of (**i**) NO_2_, (**j**) NH_3_.

The sensing performance of samples to the targeted NO_2_ and NH_3_ were investigated and the results are shown in Figure 4. Overall, the response curves of p-PANI/CNT, p-PANI and MWCNTs are upward and downward for oxidized NO_2_ and reductive NH_3_, respectively, showing the p-type sensing characteristics (Figure 4a–f) [25,26,27]. On the contrary, both n-PANI and n-PANI/CNT exhibit the n-type sensing characteristics, which are downward and upward for NO_2_ and NH_3_, respectively. The p–n-type transformation should be attributed to the dedoping of hydrochloric acid caused by heating. It is well known that hydrochloric acid is a volatile acid, which is easy to volatilize when heated, resulting in the dedoping of hydrochloric acid in PANI. Thus, the dedoping of protonic acid leads to the p–n-type transformation of PANI and its composites. As shown in Figure 4a–f, compared with p-PANI and MWCNTs, the p-PANI/CNT and n-PANI/CNT exhibited the higher sensitivity, shorter response time and recovery time, which is more clearly reflected in the column diagram in Figure S6. The responses of p-PANI/CNT to 50 ppm NO_2_ reaches 65.9, which is 6.4 and 18.3 times as much as that of p-PANI (10.3) and MWCNTs (3.6), respectively. The response time (5.2 s) of p-PANI/CNTs to 50 ppm NO_2_ is only 15.6% and 17.7% of p-PANI (33.3 s) and MWCNTs (29.4 s), respectively. For the 50 ppm NH_3_, the response of p-PANI/CNT reaches −0.975, which is 3.0 and 7.0 times as much as that of p-PANI (−0.33) and MWCNTs (−0.14), respectively. The response time (2.3 s) of p-PANI/CNT to 50 ppm NH_3_ is only 38.3% and 22.8% of p-PANI (6.0 s) and MWCNTs (10.1 s), respectively. The recovery times of p-PANI/CNT to NO_2_ and NH_3_ is only 3.2 and 4.6s, respectively, which is much shorter than that of p-PANI and MWCNTs. Obviously, the p-PANI/CNT has higher sensitivity and faster sensing speed, which should be attributed to the one-dimensional hierarchical structure. As shown in Figure 4h–j, although the response of n-PANI and n-PANI/CNT to NO_2_ decreased, they showed a higher response to NH_3_ than that of p-PANI/CNT. The response of n-PANI and n-PANI/CNT to 50 ppm of NH_3_ was up to 158.6 and 276.3, respectively. The enhanced response of n-PANI and n-PANI/CNT to NH_3_ should be attributed to the decrease of carrier concentration (Table S1) due to the dedoping of protonic acid. The doping degree of protonic acid determines the conductivity of PANI, and dedoping reduces the carrier concentration of n-PANI, thereby reducing its conductivity [11,12,13]. It is well known that the lower the carrier concentration, the deeper the charge depletion layer and the higher response [25,26,27]. Therefore, the n-PANI and n-PANI/CNT with lower conductivity show higher response for NH_3_.

**Figure 5:**
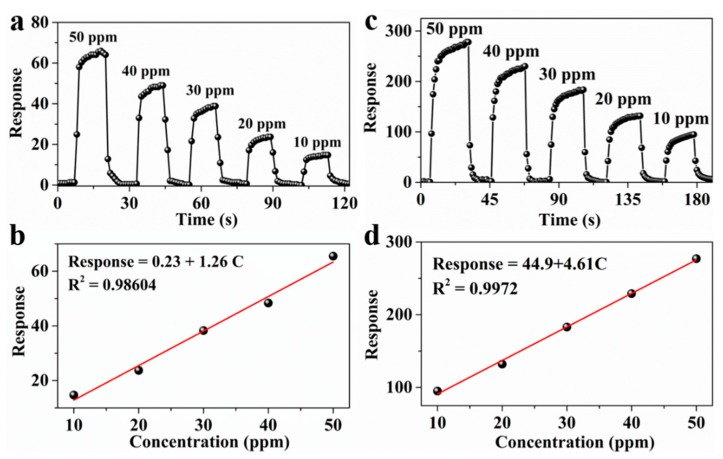
(**a**) Relation between responses of p-PANI/CNT and NO_2_ concentrations, (**b**) the fitting plots of responses Vs NO_2_ concentration, (**c**) Relation between responses of n-PANI/CNT and NH_3_ concentrations, (**d**) the fitting plots of responses Vs NH_3_ concentration.

LOD is the lowest amount of an analyte in a sample which can be detected with reasonable certainty. In all the samples, because p-PANI/CNT and n-PANI/CNT has the highest sensitivity to NO_2_ and NH_3_, respectively, the LOD of p-PANI/CNT and n-PANI/CNT to NO_2_ and NH_3_ was calculated to better evaluate the sensing performance. With the increasing NO_2_ and NH_3_ concentrations, a nearly linear dependency between the response and the concentration was observed for NO_2_ and NH_3_ (Figure 5a–d). The linear relationship shows that the adsorption of NO_2_ and NH_3_ on p-PANI/CNT and n-PANI/CNT fibers has not yet reached saturation. According to the fitting results of responses Vs concentrations in Figure 5b,d the estimated LOD (defined as LOD = 3 *S*_D_/*m*, where m is the slope of the linear part of the calibration curve and *S*_D_ is the standard deviation of noise in the response curve) for NO_2_ and NH_3_ is determined to be 16.7 and 6.5 ppb, respectively. This result is far below the exposure limit (200 ppb) set by ACGIH as mentioned above, indicating that p-PANI/CNT and n-PANI/CNT are suitable for practical application. 

To comprehensively evaluate the sensing performance of p-PANI/CNT, our sensor and the latest NO_2_ sensors were compared and the results are listed in Table 1. As can be seen from Table 1, both response time and recovery time are the shortest and the sum of response time and recovery time in a response-recovery cycle is only 8.4 s for p-PANI/CNT. Therefore, the p-PANI/CNT fibers perform better in overall response time and recovery time than the recent NO_2_ sensors, basically achieving the real-time detection of NO_2_ at RT. The LOD of MoTe_2_ to NO_2_ is as low as 0.123 ppb, but it needs ultraviolet irradiation and the response time is up to 300 s [28]. Meanwhile, although the LOD of p-PANI/CNT to NO_2_ is not the lowest, it is far below the exposure limit of NO_2_ (200 ppb) set by ACGIH. Similarly, the n-PANI/CNT with the hierarchical structure also showed a rapid and sensitive detection of NH_3_ (Table S2). The sum of response time (5.8 s) and recovery time (2.1 s) in a response-recovery cycle is only 7.9 s for n-PANI/CNT, which is the fastest response-recovery cycle in Table S2. The LOD of n-PANI/CNT to NH_3_ is as low as 6.5 ppb, which is the second lowest LOD in Table S2. The sensor based on PANI/TiO_2_ [29] display the lowest LOD (0.05 ppb), but have a response time of up to 55 s. Considering all these factors, p-PANI/CNT and n-PANI/CNT show excellent gas-sensing performance to NO_2_ and NH_3,_ respectively.

**Figure 6:**
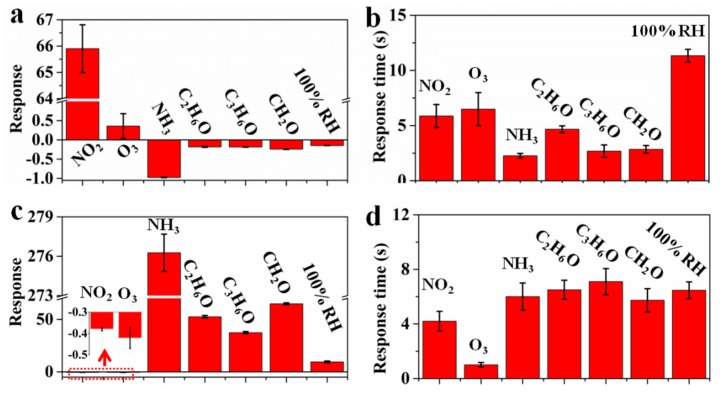
(**a**) Responses, (**b**) response time of p-PANI/CNT and (**c**) responses, (**d**) response time of n-PANI/CNT to 50 ppm of NO_2_ and NH_3_, 100 ppm of O_3_, C_2_H_6_O, C_3_H_6_O, CH_2_O and 100% RH.

Selectivity and response speed of p-PANI/CNT and n-PANI/CNT is also investigated (Figure 6a–d) and the response curves to 50 ppm of NO_2_ and NH_3_, 100 ppm of O_3_, C_2_H_6_O, C_3_H_6_O, CH_2_O and 100% RH were also recorded in Figures S7 and S8. Compared with the response (65.9) to 50 ppm of NO_2_, the response of p-PANI/CNT to 100 ppm of oxidizing O_3_ is only 0.41, which is almost negligible. The responses of p-PANI/CNT to 100 ppm of reductive C_2_H_6_O, C_3_H_6_O, CH_2_O and 100% RH are no more than −0.20, which is quite small compared with NH_3_. Due to its unique conjugate structure, PANI is particularly sensitive to acid/base doping, and then to acidic and alkaline atmosphere [21]. Therefore, p-PANI/CNT exhibits good selectivity to acidic NO_2_ and basic NH_3_. However, because of the dedoping of protonic acid, n-PANI/CNT shows exactly the opposite response to the p-PANI/CNT, displaying the n-type sensing characteristics. One can see that the responses of n-PANI/CNT to 100 ppm of C_2_H_6_O, C_3_H_6_O and CH_2_O are as high as 52.7, 37.1 and 64.8, respectively. Moreover, the response of n-PANI/CNT to 100 ppm of O_3_ exceeds that to 50 ppm of NO_2_. All these indicated that the selectivity of n-PANI/CNT to NO_2_ and NH_3_ has decreased significantly compared with p-PANI/CNT. In terms of response speed, the longest response times of p-PANI/CNT and n-PANI/CNT belong to 100% RH and C_3_H_6_O, reaching 11.8 and 7.2 s, respectively. The results reflect that both p-PANI/CNT and n-PANI/CNT have fast response speed due to the one-dimensional hierarchical structure.

**Figure 7:**
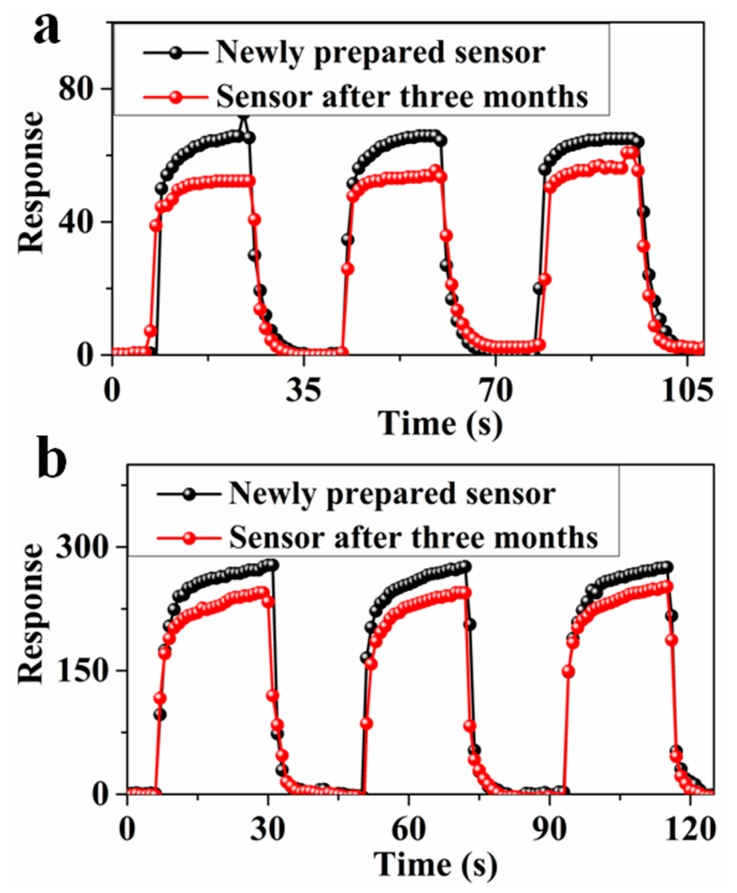
Response curves of the newly prepared sensor and sensor after three months at RT (**a**) p-PANI/CNT to 50 ppm of NO_2_, (**b**) n-PANI/CNT to 50 ppm of NH_3_.

In addition, because p-PANI/CNT and n-PANI/CNT had the highest response to NO_2_ and NH_3_, respectively, the long-term stability of p-PANI/CNT and n-PANI/CNT was tested (Figure 7). After testing the newly prepared sensors of p-PANI/CNT and n-PANI/CNT, they were kept in dust proof condition for three months at RT to test the long-term stability. Response curves of the newly prepared sensors and sensors after three months are shown in Figure 7. After three months, the responses of the p-PANI/CNT and n-PANI/CNT decreased by 19.1% and 11.2%, respectively. This indicates that the samples after heat treatment have better long-term stability.

### 3.3. Analysis of Sensing Mechanism

**Figure 8:**
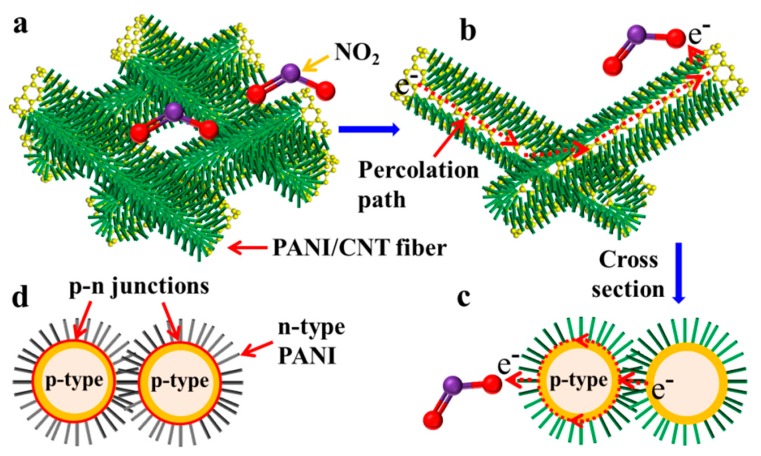
Possible sensing mechanism (**a**) sketch diagram of conductive network of hierarchical p-PANI/CNT fibers, (**b**) percolation path through conjugate interfaces of PANI and MWCNTs, (**c**) cross section of PANI/CNT fibers and (**d**) p–n heterojunction structure of hierarchical n-PANI/CNT fibers.

The high and fast response should be attributed to the following three factors, as shown in Figure 8. First, for the hierarchical p-PANI/CNT fibers, a large number of micropores exist in the conductive networks of hierarchical p-PANI/CNT fibers arranged in disorder and the hierarchical structure provides a large number of passageways (Figure 2c and Figure 8a), which provide sufficient and fast channels for the gas diffusion. It is well known that gas-sensing process occurs mainly on the surface of sensing materials [39,40]. Therefore, the effective exposure of sensing materials to the gas molecules largely determines the sensor’s sensing performance. The high permeability of hierarchical p-PANI/CNT fibers allows target gas molecules to rapidly contact with PANI fibers by the rapid diffusion through the channels, shortening the response/recovery time and enhancing the sensitivity [39,41]. Second, compared with PANI fibers, MWCNTs demonstrates the higher carrier mobilities [21,42]. Therefore, the carrier mobilities of PANI can be reinforced by constructing core–shell PANI/CNTs composites. The conjugated interfaces between PANI and MWCNTs provide the percolation path with the higher carrier mobilities (Figure 8b,c) [21,42]. Furthermore, one-dimensional structure facilitates the rapid charge transport of between PANI fibers and target gas [40,43]. When the target gas (NO_2_) is in contact with the p-PANI/CNTs, the process of charge transfer between the target gas and the hierarchical p-PANI/CNTs is accelerated due to the enhanced carrier mobility, i.e., the response/recovery time are also shortened. Third, PANI has many electron-rich amino groups [13]. For electron-deficient NO_2_, these electron-rich amino groups of PANI acting as baits induce oxidizing NO_2_ to adsorb on the surface of PANI, enhancing the sensitivity. In short, the one-dimensional structure, high permeability, enhanced carrier mobility and the interaction between amino groups and NO_2_ contributed to the sensitive and fast response. As shown in Figure 8d, because of the uniform core–shell structure of n-PANI/CNT fibers, many p–n heterojunctions are formed at the interface between n-type PANI and p-type MWCNTs. Therefore, the n-PANI/CNT fibers not only have the above advantages of p-PANI/CNT fibers, but also have a unique p–n heterojunction structure. The combination of electrons in n-type PANI and holes in p-type MWCNTs at the interface result in the lower carrier concentration, enhancing the sensitivity of n-PANI/CNT fibers [25,26,27]. In addition, the sensing performance of p-PANI/CNT also can be regulated by via surface modification to produce a cross-response sensor array and coupled with the image recognition, large data for a rapid diagnostic detection of gaseous air pollutants. The results indicate that we have succeeded in improving the gas-sensing performance of PANI and CNTs through interface engineering and harvested the synergistic effect of multiple sensing factors.

## 4. Conclusions

Two kinds of hierarchical p-PANI/CNT and n-PANI/CNT fibers were prepared. The p-PANI/CNT and n-PANI/CNT fibers performed the higher sensitivity, better reversibility, and the faster response and recovery than the PANI and MWCNTs. The p-PANI/CNT and n-PANI/CNT fibers displayed the better sensitivity to NH_3_ and NO_2_, respectively. Both p-PANI/CNT and n-PANI/CNT showed the real-time response ability. The estimated LOD for NO_2_ and NH_3_ is determined to be 19.6 and 6.5 ppb, respectively. It was found that one-dimensional morphology, hierarchical structures the enhanced carrier mobility and p–n heterojunctions contributed to the higher sensitivity and faster responses. This work also looks forward to the development prospects of regulable, cost-effective and high-performance PANI/CNT-based sensors in the context of IoT and the potentials of interface engineering in improving gas-sensing performance.

## Figures and Tables

**Table 1:** Comparison of the chemiresistive sensor based on hierarchical p-PANI/CNT fibers and other sensors to NO_2_.

Samples	Working Temperature	Concentration of NO_2_ (ppm)	Response	Response/Recovery Time	LOD (ppb)	Ref.
CVD-graphene	200 °C	100 ppb	∼4%	50/50 min	--	[30]
Graphene	150 °C	5 ppm	7%	10/30 min	--	[31]
Graphene/MoS_2_	150 °C	5 ppm	7%	5/> 30 min	--	[32]
PbS CQDs	RT	50	21.7	12/37s	84	[10]
Graphene	--	50	24.7	∼500/2500s	3600	[33]
RGO	RT	5	11.5%	420/1680s	1000	[34]
MoTe_2_	RT	0.02	18%	300/120s	0.123	[28]
ZnO/m-SWCNT	RT	2.5	52%	208/> 208s	2500	[35]
ZnO NR	200 °C	1	41%	48/180s	1000	[36]
RGO/SnO_2_	50 °C	5	3.31	135/200s	500	[37]
rGO/PNFs	RT	0.5	∼68%	∼150/300s	17.5	[5]
CVD-graphene	RT	5	12%	1000/> 1 h	--	[38]
p-PANI/CNT	RT	50	65.9	5.2/3.2s	16.7	This work

## Supplementary Materials

The following are available online at https://www.mdpi.com/1424-8220/20/1/149/s1, Figure S1. Zeta potentials of (a) MWCNTs, (b) PANI, (c) MWCNTs and PANI and (d) MWCNTs and PANI after 3 min, Figure S2. Schematic diagram for the synthesis of PANI/CNT fibers using MWCNTs via interface interaction, Figure S3. TEM image of fibrous PANI, Figure S4. X-ray photoelectron spectroscopy (XPS) of samples, Figure S5. The plot of transformed Kubelka-Munk function versus the energy of light (a) p-PANI, p-PANI/CNT, (b) n-PANI, n-PANI/CNT, Figure S6. (a) Response times, (b) recovery times of p-PANI/CNT, p-PANI, MWCNTs, n-PANI and n-PANI/CNT to 50 ppm of NO_2_ and NH_3_, Figure S7. Response curves of p-PANI/CNT to 100 ppm of (a) O_3_, (b) C_2_H_6_O, (c) C_3_H_6_O, (d) CH_2_O and (e) 100% RH, Figure S8. Response curves of n-PANI/CNTs to 100 ppm of (a) O_3_, (b) C_2_H_6_O, (c) C_3_H_6_O, (d) CH_2_O and (e) 100% RH, Table S1. Average conductivity of MWCNTs, p-PANI, p-PANI/CNT, n-PANI and n-PANI/CNT, Table S2. Comparison of the chemiresistive sensor based on hierarchical p-PANI/CNTs fibers and other sensors to NH_3_.
