# Supplementary material for: High-Performance Gas Sensor of Polyaniline/Carbon Nanotube Composites Promoted by Interface Engineering
Source: Sensors (Basel). 2019 Dec 25;20(1):149. doi: 10.3390/s20010149 (PMC6982802; doi:10.3390/s20010149)
Supplement: Supplementary file 1 [file sensors-20-00149-s001.pdf]

Supplementary Material

High-Performance Gas Sensor of Polyaniline/Carbon Nanotube Composites Promoted by Interface Engineering

WeiyuZhang,[a] Shuai Cao,[a] ZhaofengWu,[a,b*] Min Zhang,[a] YaliCao,[b] Jixi Guo,[b] FuruZhong,[b] HaimingDuan,[a*] DianzengJia[b*]

[a] School of Physics Science and Technology, Xinjiang University, Urumqi, Xinjiang 830046, China

[b] Key Laboratory of Energy Materials Chemistry, Ministry of Education, Key Laboratory of Advanced Functional Materials, Xinjiang University, Urumqi, Xinjiang 830046, China

* Correspondence: wuzf@xju.edu.cn, dhm@xju.edu.cn, jdz@xju.edu.cn; Tel.: +86-991-858-2401

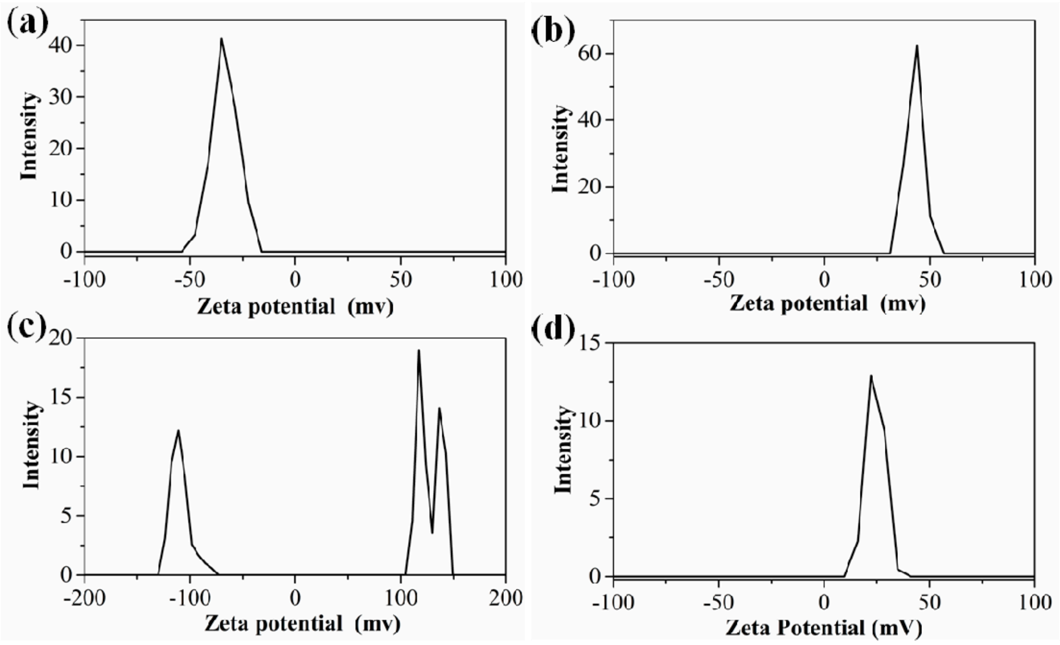

Figure 1. Zeta potentials of (a) MWCNTs, (b) PANI, (c) MWCNTs and PANI and (d) MWCNTs and PANI after 3 min.

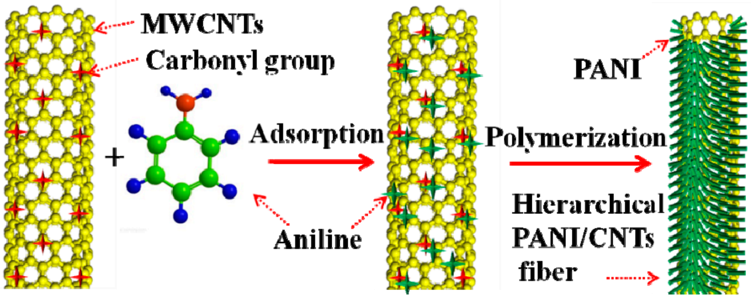

Figure 2. Schematic diagram for the synthesis of PANI/CNT fibers using MWCNTs via interface interaction.

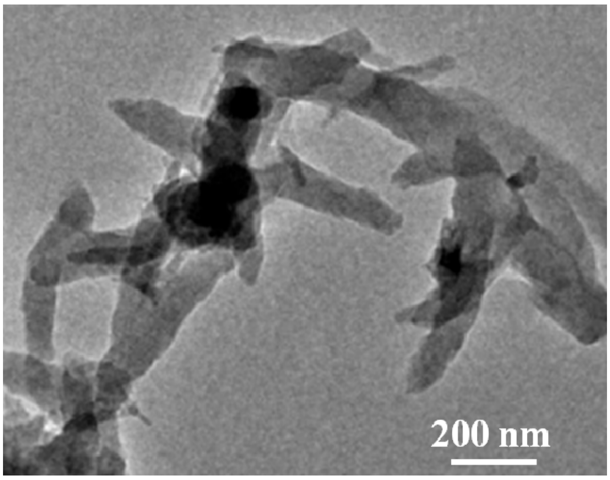

Figure 3. TEM image of fibrous PANI.

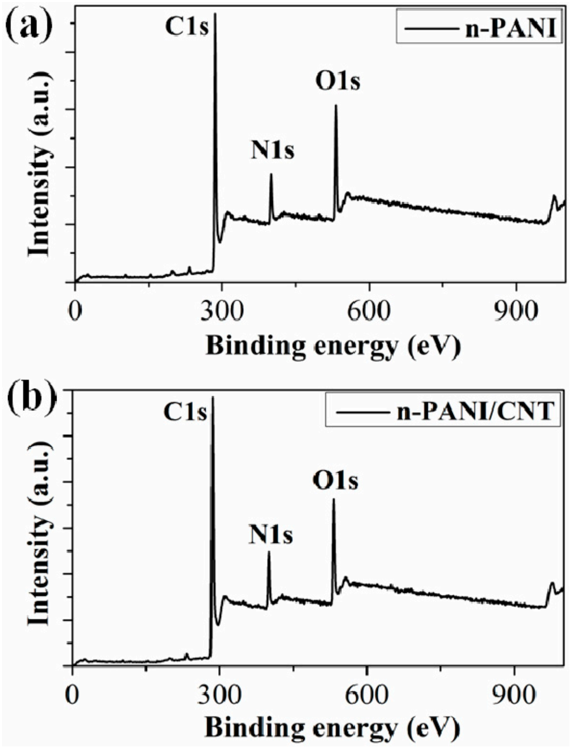

Figure 4. X-ray photoelectron spectroscopy (XPS) of samples.

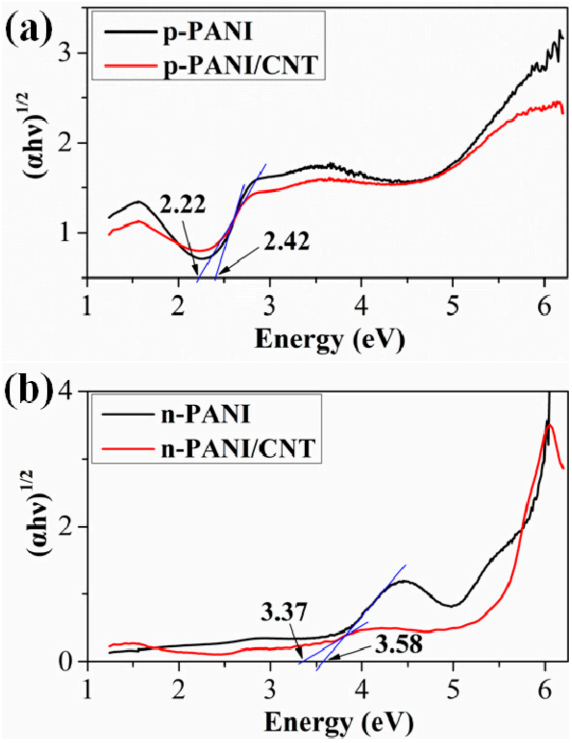

Figure 5. The plot of transformed Kubelka-Munk function versus the energy of light (a) p-PANI, p-PANI/CNT, (b) n-PANI, n-PANI/CNT.

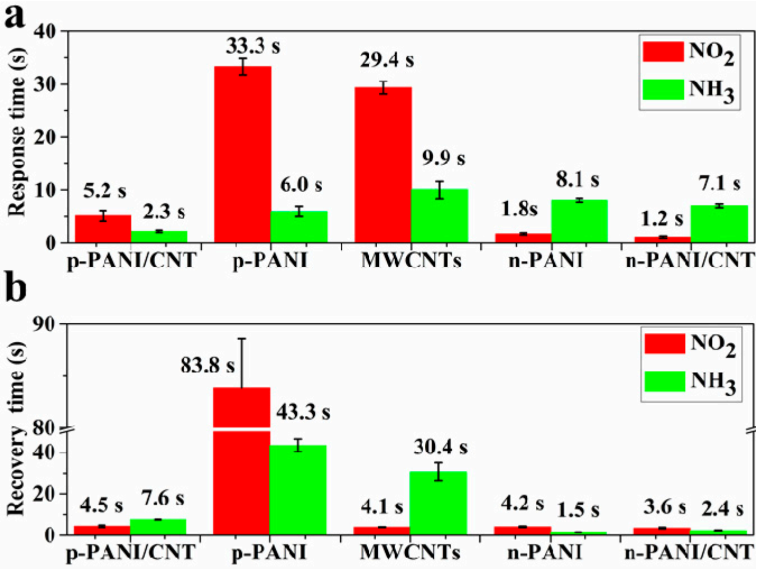

Figure 6. (a) Response times, (b) recovery times of p-PANI/CNT, p-PANI, MWCNTs, n-PANI and n-PANI/CNT to 50 ppm of NO_2 and NH_3.

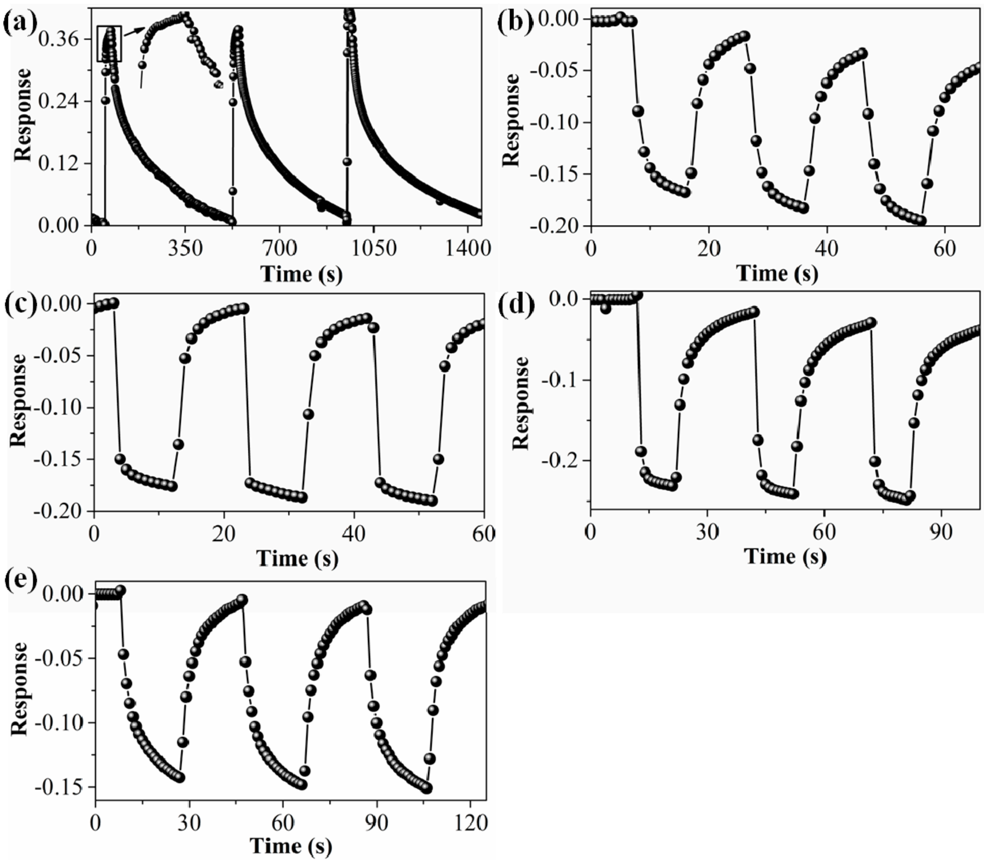

Figure 7. Response curves of p-PANI/CNT to 100 ppm of (a) O_3, (b) C_2H_6O, (c) C_3H_6O, (d) CH_2O and (e) 100% RH.

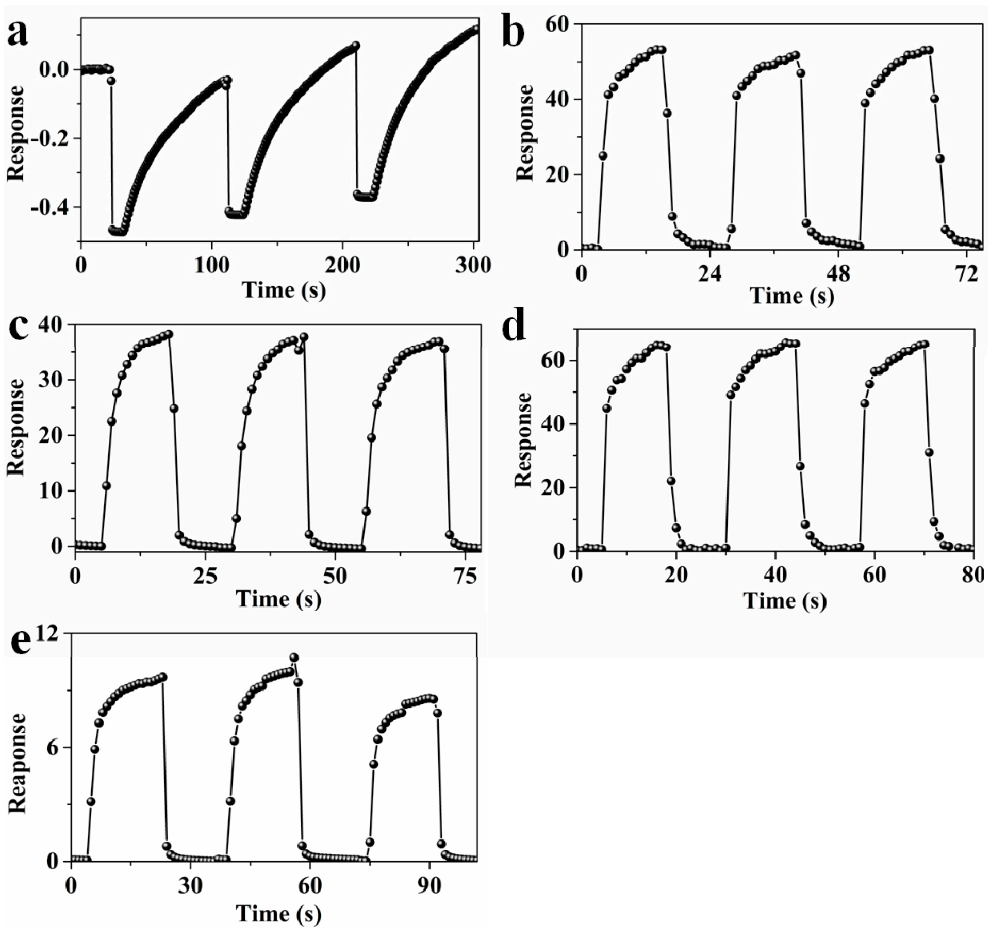

Figure 8. Response curves of n-PANI/CNTs to 100 ppm of (a) O_3, (b) C_2H_6O, (c) C_3H_6O, (d) CH_2O and (e) 100% RH.

Table 1. Average conductivity of MWCNTs, p-PANI, p-PANI/CNT, n-PANI and n-PANI/CNT.

Samples	Conductivity 1 (s/cm)	Conductivity2(s/cm)	Conductivity 3 (s/cm)	Average conductivity (s/cm)
MWCNTs	99.8	100.2	101.1	100.34
p-PANI	1.2E-3	1.5E-3	1.8E-3	1.5E-3
p-PANI/CNT	9.8E-3	8.9E-3	9.1E-3	9.3E-3
n-PANI	8.9E-6	8.2E-6	9.1E-6	8.7E-6
n-PANI/CNT	1.2E-6	1.8E-6	2.1E-6	1.7E-6

Table 2. Comparison of the chemiresistive sensor based on hierarchical p-PANI/CNTs fibers and other sensors to NH_3.

Samples	Concentration of NH_3 (ppm)	Response	Response/ Recovery time	LOD (ppb)	Ref.
2%Fe_2O_3-ZnO	0.4	10^4	20/20s	--	[1]
SnO_2/Pt	48	13	◎10/30s	--	[2]
Mn-ZnO	100	28.5	4/0.8s	--	[3]
Ag-ZnO	10	29.5	13/20s	--	[4]
SnO_2-Pt	450	25.7	◎1/59s	--	[5]
cobalt-ZnO	100	2.87	3.48/19s	15000	[6]
SnO_2-rGO	200	1.3	8/13s	--	[7]
PSS doped PANI	100	125	20/20s	100	[8]
PANI/TiO_2	0.0002	1.8%	◎55s /--	0.05	[9]
PANI/Mn_2O_3	0.3	30	10/80s	25	[10]
PANI/MWCNT	100	120%	50/200s	200	[11]
PANI/MWCNT	10	600%	100/300s	200	[12]
PANI/rGO	0.1	49	18/36s	100	[13]
PANI/SnO_2	93	5	2/60s	23000	[14]
PANI/Ag	10	850	240/60s	1000	[15]
PANI/Cu	50	85%	7s/160s	1000	[16]
PANI/ZnO	100	27%	40/300s	10000	[17]
n-PNAI/CNT	50	276.3	5.8/2.1s	6.5	This work

References

1. Tang, H.; Yan, M.; Zhang, H.; Li, S.; Ma, X.; Wang, M.; Yang, D. A selective NH3 gas sensor based on Fe2O3–ZnO nanocomposites at room temperature. Sens. Actuators, B. **2006,** *114*, 910-915.
2. Anisimov, O. V.; Maksimova, N. K.; Chernikov, E. V.; Sevastyanov, E. Y.; Sergeychenko, N. V. *Sensitivity to NH3 of SnO2 thin films prepared by magnetron sputtering*, 2009 International Siberian Conference on Control and Communications, 27-28 March 2009; 2009; 10689253
3. Sankar Ganesh, R.; Durgadevi, E.; Navaneethan, M.; Patil, V. L.; Ponnusamy, S.; Muthamizhchelvan, C.; Kawasaki, S.; Patil, P. S.; Hayakawa, Y. Low temperature ammonia gas sensor based on Mn-doped ZnO nanoparticle decorated microspheres. *J Alloy Compd.* **2017,** *721*, 182-190.
4. R, S. G.; M, N.; Patil, V. L.; S, P.; C, M.; Kawasaki, S.; Patil, P. S.; Hayakawa, Y. Sensitivity enhancement of ammonia gas sensor based on Ag/ZnO flower and nanoellipsoids at low temperature. Sens. Actuators, B. 2018, 255, 672-683.

5. Shahabuddin, M.; Sharma, A.; Kumar, J.; Tomar, M.; Umar, A.; Gupta, V. Metal clusters activated SnO2 thin film for low level detection of NH3 gas. Sens. Actuators, B. 2014, 194, 410-418.
6. Mani, G. K.; Rayappan, J. B. B., A highly selective and wide range ammonia sensor—Nanostructured ZnO:Co thin film. Mat. Sci. Eng. B. 2015, 191, 41-50.
7. Chen, Y.; Zhang, W.; Wu, Q., A highly sensitive room-temperature sensing material for NH3: SnO2-nanorods coupled by rGO. Sens. Actuators, B. 2017, 242, 1216-1226.
8. Jang, J.; Ha, J.; Cho, J. Fabrication of water-dispersible polyaniline-poly (4-styrenesulfonate) nanoparticles for inkjet-printed chemical-sensor applications. Adv. Mater. 2007, 19, 1772-1775.
9. Gong, J.; Li, Y. H.; Hu, Z. S.; Zhou, Z. Z.; Deng, Y. L. Ultrasensitive NH3 Gas Sensor from Polyaniline Nanograin Enchased TiO2 Fibers. J. Phys. Chem. C. 2010, 114, 9970-9974.
10. Li, Y.; Gong, J.; He, G.; Deng, Y. Synthesis of polyaniline nanotubes using Mn2O3 nanofibers as oxidant and their ammonia sensing properties. Synthetic. Met. 2011, 161, 56-61.
11. He, L.; Jia, Y.; Meng, F.; Li, M.; Liu, J. Gas sensors for ammonia detection based on polyaniline-coated multi-wall carbon nanotubes. Mat. Sci. Eng. B. 2009, 163, 76-81.
12. Xue, L.; Wang, W.; Guo, Y.; Liu, G.; Wan, P., Flexible polyaniline/carbon nanotube nanocomposite film-based electronic gas sensors. Sens. Actuators, B. 2017, 244, 47-53.
13. Guo, Y.; Wang, T.; Chen, F.; Sun, X.; Li, X.; Yu, Z.; Wan, P.; Chen, X. Hierarchical graphene–polyaniline nanocomposite films for high-performance flexible electronic gas sensors. Nanoscale. 2016, 8, 12073-12080.
14. Tai, H.; Jiang, Y.; Xie, G.; Yu, J. Preparation, Characterization and Comparative NH3-sensing Characteristic Studies of PANI/inorganic Oxides Nanocomposite Thin Films. J. Mater. Sci. Technol. 2010, 26, 605-613.
15. Athawale, A. A.; Katre, P. P. In Ag dispersed conducting polyaniline nanocomposite as a selective sensor for ammonia, Journal of Metastable and Nanocrystalline Materials. Trans Tech Publ, 2005, 323-326.
16. Patil, U. V.; Ramgir, N. S.; Karmakar, N.; Bhogale, A.; Debnath, A. K.; Aswal, D. K.; Gupta, S. K.; Kothari, D. C. Room temperature ammonia sensor based on copper nanoparticle intercalated polyaniline nanocomposite thin films. Appl. Surf. Sci. 2015, 339, 69-74.
17. Patil, S. L.; Chougule, M. A.; Sen, S.; Patil, V. B. Measurements on room temperature gas sensing properties of CSA doped polyaniline–ZnO nanocomposites. Measurement. 2012, 45, 243-249.
